# Immunotherapy for High-Risk Neuroblastoma: Management of Side Effects and Complications

**DOI:** 10.6004/jadpro.2017.8.1.4

**Published:** 2017-01-01

**Authors:** Erin Armideo, Colleen Callahan, Lara Madonia

**Affiliations:** The Children’s Hospital of Philadelphia, Philadelphia, Pennsylvania

## Abstract

Despite more intensive therapy, high-risk neuroblastoma continues to be a challenging disease to treat. Postconsolidation immunotherapy has been studied for many years and has proven to be effective in clinical trials. Immunotherapy has become the standard of care for patients with high-risk disease, and many institutions across the country are providing this therapy. The care of these patients is complex and often associated with many side effects. The purpose of this article is to review the most common side effects seen in clinical practice and examine their management. Furthermore, this article will discuss the need for a consistent and educated multidisciplinary front-line team to care for these patients, with advanced practitioners playing a lead role to provide the care and attention needed for this patient population.

Neuroblastoma is the most common extracranial solid tumor in children. Approximately 800 children are diagnosed with neuroblastoma each year in the United States (Ward, DeSantis, Robbins, Kohler, & Jemal, 2014). Risk stratification in children with neuroblastoma is not only based on disease stage but also the age of the child at diagnosis and the biologic and histologic characteristics of the tumor.

The International Neuroblastoma Risk Group Staging system (INRG) is the most commonly used system for staging neuroblastoma (Cohn et al., 2009). The International Neuroblastoma Pathology Classification (INPC) system is used to designate whether the tumor has favorable or unfavorable histologic features (Shimada et al., 1999). The status of the *MYCN* oncogene is important in risk stratification as well, as amplification of this gene within tumor cells is associated with a poor prognosis (Brodeur, Seeger, Schwab, Varmus, & Bishop, 1984).

This article will focus on those patients who have high-risk disease, including all patients over 18 months of age at diagnosis who have metastatic (INRG stage M) disease. It also includes subsets of patients with localized disease and infants with metastatic disease in a limited distribution (INRG stage MS disease) whose tumors have amplification of the *MYCN* gene. Of all patients diagnosed with neuroblastoma, approximately half present with high-risk disease (Irwin & Park, 2015). Despite intensive therapy, less than half of patients with high-risk disease remain relapse free at 3 years from diagnosis (Kreissman et al., 2013).

Patients with high-risk neuroblastoma receive intensive multimodality therapy. These therapies can be broken down into phases including induction, consolidation, and postconsolidation therapy. The induction phase includes multiagent chemotherapy and surgery. The goal of this phase of therapy is to reduce the amount of disease not only at the primary site but also at metastatic sites. The consolidation phase includes myeloablative chemotherapy with stem cell rescue followed by external-beam radiation therapy, which is directed at the primary tumor site and other persistent sites of disease. The postconsolidation or maintenance phase of therapy includes immunotherapy and a differentiating agent. The goal of this phase is to eliminate any residual tumor cells that may exist following maximally intensive treatment. Postconsolidation immunotherapy has been proven to be effective in a randomized clinical trial. The complications and side effects from this treatment can be challenging, and therefore, the remainder of this article will focus on this phase of therapy (Yu et al., 2010). Management of some of the most common complications will be emphasized.

## POSTCONSOLIDATION THERAPY: OVERVIEW OF DRUGS

The postconsolidation phase of high-risk neuroblastoma therapy consists of four drugs. They include dinutuximab (Unituxin; formerly known as ch14.18), aldesleukin (Proleukin; also known as IL-2), granulocyte macrophage colony-stimulating factor (GM-CSF), and isotretinoin. This therapy received approval by the US Food and Drug Administration (FDA) in March 2015 (US FDA, 2015) and is approved as part of first-line therapy for pediatric patients with high risk neuroblastoma (US FDA, 2015).

Dinutuximab is a monoclonal antibody that targets GD2, which is a disialoganglioside expressed on the surface of neuroblastoma cells (Schulz et al., 1984). The effect of this monoclonal antibody is augmented by two other agents, GM-CSF and IL-2. The purpose of giving these agents in combination with dinutuximab is to stimulate the immune response. Once the dinutuximab recognizes the GD2 antigen on the neuroblastoma cells, the cells of the immune system are then able to lyse the target cells. Both GM-CSF and IL-2 are given to enhance the antitumor effect; IL-2 works to stimulate natural killer cells, and GM-CSF activates granulocyte and macrophage cytotoxicity (Yu et al., 2010).

The fourth agent that is given during immunotherapy is isotretinoin. Isotretinoin is a differentiating agent that induces the maturation of neuroblastoma cells (Greengard, Hill-Kayser, & Bagatell, 2013).

## OVERALL TREATMENT SCHEMA

Dinutuximab is currently given as part of a multidrug regimen that consists of 6 cycles given over the course of approximately 6 months, as extensively studied in the Children’s Oncology Group (COG) study ANBL0032.

Courses 1, 3, and 5 consist of GM-CSF, dinutuximab, and isotretinoin. The GM-CSF is given for 14 days and typically starts at home 3 days prior to the start of dinutuximab. Dinutuximab is given for 4 days in the inpatient setting. Isotretinoin is given orally and is started 10 days after the start of GM-CSF. Isotretinoin administration continues for 14 days. Cycles 2 and 4 consist of IL-2, dinutuximab, and isotretinoin. Low-dose (3 million IU/m²) IL-2 is given as a continuous infusion over 4 days; it is given on days 0 to 3 of cycle 2 and 4 and is administered either in the inpatient or outpatient setting, based on institutional practice and the availability of home health support. One week after the start of low-dose IL-2, a higher dose of IL-2 (4.5 million IU/m²) is given continuously over 4 days along with dinutuximab. This combination is delivered in the inpatient setting. During cycles 2 and 4, isotretinoin begins 14 days after the start of the treatment cycle and continues for 14 days. Cycle 6 consists of isotretinoin alone. (See Figure for the overall schema.)

**Figure:**
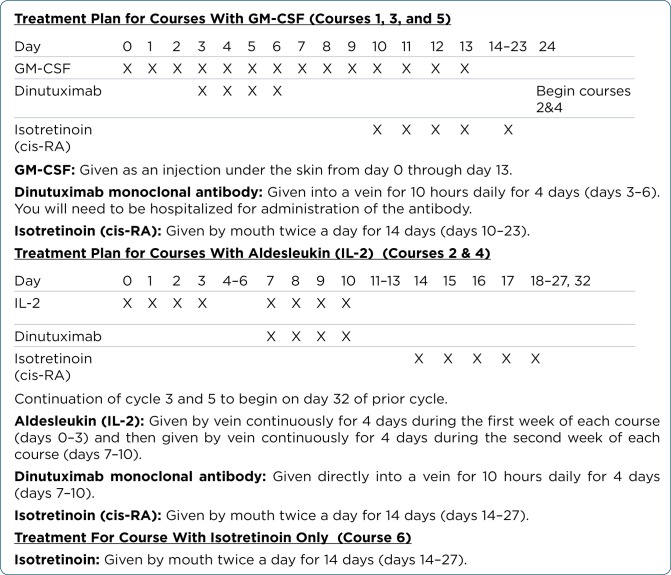
Overall Schema for Treatment With Dinutuximab. cis-RA = cis-retinoic acid; GM-CSF = granulocyte macrophage colony-stimulating factor; IL-2 = interleukin-2.

A complete disease evaluation is performed before immunotherapy is initiated, and although a repeat disease evaluation was not mandatory on COG ANBL0032, it is strongly recommended. The toxicity associated with this therapy is nontrivial, and if a patient has had disease progression on therapy, it is important to move on to other therapeutic options rather than continuing to deliver treatment known to have significant side effects.

A complete disease evaluation is also performed at the end of therapy and permits establishment of a new baseline, particularly for patients with residual or evolving metaiodobenzylguanidine (MIBG)-avid bone lesions. A substance that localizes to certain tissues including neuroblastoma, MIBG is combined with radioactive iodine and then injected into the patient. The MIBG scans are particularly helpful in evaluating response. The MIBG scan also helps to identify sites of neuroblastoma in the patient. Based on our experience at The Children’s Hospital of Philadelphia, we now include a computed tomography (CT) scan of the brain with disease evaluations during immunotherapy, as we have seen more and more central nervous system (CNS) recurrences since the addition of this therapy.

**Intravenous Access**

Intravenous access is a critical consideration as immunotherapy is planned. Care is complex during the administration of dinutuximab, and multiple intravenous (IV) medications and infusions are needed. Dinutuximab, IL-2, pain medications, fluids, antihistamines, and antibiotics are given intravenously, and most young patients benefit from having two lumens for central access. Some children, particularly older children, may be able to tolerate therapy with a single-lumen central line and a peripheral IV line for access. If single-lumen access is considered, the patient should have his or her veins evaluated to ensure that peripheral access is sufficient.

## ADMINISTRATION OF DINUTUXIMAB

Based on the experience in ANBL0032, dinutuximab is infused over a minimum of 10 hours. Vital signs should be taken every 15 minutes for the first hour of the infusion and then hourly until the infusion is complete. Initially, the antibody infusion is run at a 50% rate for 30 minutes. If the patient is tolerating this rate and vital signs are stable, the antibody is then increased to a rate to complete the infusion in 10 hours. The infusion rate may be slowed down if the patient experiences complications (as described below). The infusion must finish within 20 hours from the start due to concerns about drug stability beyond this point. If the patient has a pause or decrease in the infusion rate for related side effects, the infusion should be turned off 20 hours from the start of the infusion. If a full dose was not received, the missed dose should not be redosed.

**Side Effects**

The administration of dinutuximab is associated with significant side effects. Effects are generally acute in nature and usually resolve once treatment ends. Some of the most frequently seen side effects include neuropathic pain (52%), fever (39%), infection (39%), hypokalemia (35%), hypersensitivity reaction (25%), capillary leak syndrome (23%), hyponatremia (23%), transaminitis (elevated alanine transaminase [ALT] 23%; elevated aspartate transaminase [AST] 10%), gastrointestinal side effects (nausea, vomiting, diarrhea; 22%), hypotension (18%), hypoxia (13%), and urticaria (13%; Yu et al., 2010).

## MANAGEMENT OF COMMON SIDE EFFECTS

**Pain**

As discussed previously, dinutuximab targets the disialoganglioside GD2 on the surface of neuroblastoma cells; GD2 is also expressed on neurons and peripheral sensory nerve fibers. For this reason, patients experience neuropathic pain during dinutuximab infusions. The most frequent site of pain is the abdomen; however, patients may experience pain anywhere throughout the body (Yu et al., 2010).

In anticipation of neuropathic pain, all patients receive a required IV bolus of a narcotic pain medication and are started on a patient-controlled analgesia (PCA) infusion approximately 30 minutes prior to starting dinutuximab each day. Pain is seen most frequently during course 1, and close monitoring and frequent titrations of the IV pain medications are critical (Yu et al., 2010). A thorough discussion of the patient’s previous experience with pain and medications used to treat pain can be very helpful in determining the starting doses for the PCA. Doses that were effective in the postoperative setting or doses that provided relief from mucositis pain during autologous transplant may provide a good starting point for the PCA used during treatment with dinutuximab. Pain is usually better controlled in subsequent cycles, potentially due to tachyphylaxis but also because patient-specific narcotic dosing requirements can be established during cycle 1, and dosing needed during subsequent cycles is more predictable.

The majority of patients are started on a morphine PCA. However, patients with morphine allergies and those who do not receive adequate pain control with morphine may benefit from a hydromorphone or fentanyl infusion. Due to the lack of information about the compatibility of other narcotics with dinutuximab and IL-2, patients receiving hydromorphone or fentanyl may require additional IV access.

In most patients, pain subsides or resolves after the completion of each day’s antibody infusion. In our practice at the Children’s Hospital of Philadelphia, the PCA is turned off 2 hours after the completion of the dinutuximab infusion. Most patients tolerate this well and have no pain once the infusion is off, and discontinuous use of the PCA appears to decrease the incidence of narcotic withdrawal.

Occasionally, patients will experience pain after the completion of dinutuximab infusion, and so a continuous narcotic infusion is required. Continuous narcotic infusions may be associated with more significant constipation, itching, urinary retention, or nausea and vomiting. Patients who receive large doses of narcotics while receiving antibody therapy may require a narcotic wean at home to prevent withdrawal. It is rare for patients to experience pain for a few days after the completion of antibody therapy, and they may benefit from scheduled doses of oral narcotics at home.

In addition to PCA, gabapentin has also been effective in decreasing neuropathic pain and may permit the use of lower doses of narcotics. The optimal effect of gabapentin is not observed immediately, and therefore it is recommended that this drug be started approximately 1 week prior to the start of immunotherapy and titrated up to a full dose over the course of several days. For the same reason, gabapentin should not be started and stopped each cycle but rather taken continuously during the five courses of immunotherapy.

In our experience, most patients have achieved adequate pain control with gabapentin and a titrated dose of narcotics. However, in rare cases, a patient may experience unbearable pain, even with high doses of narcotics. These patients are at risk for respiratory depression and may benefit from an intensive care unit setting for closer monitoring and dose escalation as needed. Some centers use lidocaine infusions for patients whose pain cannot be controlled with narcotics. In exceedingly rare cases, a dissociative agent, such as ketamine, can be used to address truly refractory pain.

**Fever**

Due to the body’s immune response during dinutuximab-based therapy, fevers are an expected side effect. Fevers may occur at any time during dinutuximab infusion and even for hours after completion of the infusion. Since most patients receiving immunotherapy have a central line, institutional guidelines regarding fever in an immunocompromised host with an indwelling catheter should be followed. In our institution, blood cultures are sent to rule out infection after the patient experiences his or her first fever during dinutuximab treatment. A broad-spectrum antibiotic that provides prolonged coverage is given. If a child appears ill or has developed neutropenia, different antibiotics may be chosen.

Because limited compatibility data are available regarding the compatibility of most antibiotics and dinutuximab, the antibody or cytokine infusion is often stopped for a short period while the antibiotic is given through the central line. The infusion can then be resumed at the full rate and does not need to be escalated again.

Because fever is an expected side effect of antibody therapy, acetaminophen is often given as a premedication before dinutuximab is administered. Despite this intervention, patients often still have high fevers, which can sometimes be alarming to both nurses, the patient, and his or her family. If the fever persists despite the use of acetaminophen, ibuprofen may be used if in accordance with institutional practice and if the patient’s platelet count permits its use. The addition of ibuprofen is often effective. In our experience, virtually all patients experience fever during immunotherapy. Educating the patient, family, and staff about fever prior to beginning dinutuximab therapy has been helpful. Anticipation of a high fever can often lessen the fears and concerns of the patient and family.

**Hypersensitivity Reaction**

Hypersensitivity reactions may be attributable to the toxic effects of dinutuximab, IL-2, or a combination of both. Hypersensitivity reactions can be seen during any dinutuximab-containing cycle but were noted to be more frequent during IL-2–containing courses (courses 2 and 4; Yu et al., 2010). Patients may experience reactions such as mild dyspnea, wheezing, flushing, rash, or urticaria. Approximately one-third of patients treated with urticaria at our center experienced hypersensitivity reactions. Mild symptoms may quickly resolve, but in rare cases they may escalate to more serious reactions, such as bronchospasm, edema, angioedema, hypotension, or anaphylaxis.

To prevent such reactions, patients receiving dinutuximab are given diphenhydramine (0.5–1 mg/kg every 4–6 hours around the clock) starting 30 minutes prior to the infusion and every 6 hours thereafter. Patients who experience mild reactions may benefit from the addition of other antihistamines, such as cetirizine, ranitidine, or hydroxyzine, or an increase in the frequency of scheduled diphenhydramine.

If a patient is having a mild allergic reaction, the rate of dinutuximab should be decreased by half and the cytokine (GM-CSF or IL-2) may continue at its full rate. During this time, patients require close monitoring and serial exams. After the resolution of symptoms, the infusion of dinutuximab may return to its full rate.

Patients who experience severe allergic reactions require immediate attention, and both infusions should be held immediately. Oxygen and albuterol may be indicated, and additional doses of diphenhydramine or other antihistamines may be given on an urgent or emergent basis. Epinephrine should be given immediately if there are signs of significant airway compromise or if airway issues are accompanied by cardiovascular collapse. Due to the concern that steroids may diminish the effect of immunotherapy, the use of steroids is reserved for those patients who are experiencing true anaphylaxis with cardiorespiratory collapse or for those who are unresponsive to two or more doses of epinephrine. Our team has worked closely with the rapid response team and intensive care personnel to explain the purpose of immunotherapy and to increase awareness of ways to avoid systemic steroids for reactions that can potentially be managed with other agents. Among 75 patients treated since initiation of this effort at our center, only 1 has required systemic steroids for a severe allergic reaction.

If hypersensitivity or allergic symptoms resolve promptly following the previously mentioned interventions, the patient may resume dinutuximab at half the original infusion rate without resumption of cytokine during the same day. If the patient is stable the following day, cytokines may be resumed.

**Capillary Leak and Hypotension**

Capillary leak has been documented to occur more frequently during IL-2–containing courses than during GM-CSF–containing courses (Yu et al., 2010). Symptoms may include hypotension, generalized edema with or without pulmonary edema and ascites, decreased intravascular volume, and decreased urine output. Due to the risk of fluid shifts, patients are weighed twice daily, intake and output are carefully documented, and IV fluid rates are titrated based on their status. Hyponatremia may occur as a result of capillary leak syndrome; however, this electrolyte abnormality is generally not severe enough to require administration of hypertonic saline. Electrolytes should be monitored daily or more frequently if needed.

To ensure euvolemia at the start of each infusion of dinutuximab, patients should receive a bolus of normal saline daily prior to starting the antibody infusion. Third spacing with capillary leak may result in significant weight gain, and peripheral edema may be impressive; however, the use of diuretics results in intravascular volume depletion and will often worsen the risk of hypotension. Furthermore, diuretics may augment electrolyte imbalances and therefore should be used cautiously. Having a consistent front-line clinician team that is educated and experienced in determining each patient’s intravascular volume with the expected fluid shifts during this therapy is key to the successful administration of therapy. Patients who experience severe capillary leak often benefit from packed red blood cells or albumin to replete the intravascular volume. Frequent physical exams are required to monitor for the presence of crackles on lung exam, an S3 on cardiac exam, or hepatosplenomegaly, all of which are signs of significant fluid overload.

Capillary leak and resulting hypotension occur frequently enough in patients receiving dinutuximab therapy that front-line caregivers must be prepared to address this complication rapidly and consistently. In our experience, a clearly outlined algorithm for hypotension management, which was developed in collaboration with all team members and is regularly reviewed by providers, has proven helpful in decreasing the need for intensive care support.

Approaches to management of hypotension may vary slightly from center to center, but general principles of management broadly apply. If a patient experiences hypotension during dinutuximab and/or IL-2 infusion, both infusions should be immediately stopped until hypotension resolves. Volume resuscitation according to institutional guidelines should commence as rapidly as possible. Consultation with a rapid response team may be considered for patients requiring multiple volume boluses. Because narcotics may contribute to decreases in blood pressure, it may be beneficial to stop the continuous narcotic infusion until the hypotension resolves.

Although normal saline is the most widely available isotonic fluid on oncology units and is generally effective for patients experiencing dinutuximab-associated hypotension, the use of colloids (albumin or blood products) can be beneficial in patients unresponsive to initial fluid resuscitation in specific circumstances. Patients with a hemoglobin < 8 g/dL or an albumin < 3.0 g/dL have benefited from transfusions prior to restarting dinutuximab infusion. Prompt recognition of hypotension, rapid discontinuation of inciting infusion, and aggressive volume resuscitation permit resumption of dinutuximab therapy in the vast majority of patients who experience this toxicity.

Although approximately 50% of patients treated with dinutuximab in our institution have been identified as having any degree of capillary leak, less than 10% have developed clinically significant hypotension (grade 3 per Common Terminology Criteria for Adverse Events), and less than 3% required transfer to the intensive care unit for hypotension. In our patient population, rapid recognition and treatment of hypotension have minimized the need for vasopressors. On the rare occasion that a patient experiences fluid-refractory hypotension, epinephrine or norepinephrine is administered and titrated to effect. Due to concerns regarding the potential for dopamine to induce an increase in prolactin levels, which can theoretically inhibit T-cell function, dopamine is not the preferred vasopressor for use in patients with high-risk neuroblastoma receiving immunotherapy.

Upon resolution of dinutuximab-related hypotension, the drug infusion can be restarted at 50% rate. For patients receiving dinutuximab and GM-CSF (cycles 1, 3, and 5), dinutuximab may be resumed at the full rate if the patient’s blood pressure remains stable for 2 hours at the lower rate. For patients receiving IL-2–containing cycles (cycles 2 and 4), if the blood pressure is stable for 2 hours after resumption of dinutuximab at half rate, IL-2 may be restarted. In the absence of recurrent hypotension in the subsequent 2 hours, dinutuximab may be increased to the full rate.

**Hypoxia**

Hypoxia may be experienced by patients who develop hypersensitivity reactions, capillary leak, or oversedation due to narcotics for uncontrolled pain. Continuous pulse oximetry monitoring is required during dinutuximab treatment. Oxygen supplementation may be necessary to maintain appropriate oxygenation. Since the nurse practitioner team at our institution has become the primary front-line team caring for these patients, we have not had any patients experience dose-limiting hypoxia related to dinutuximab therapy.

**Gastrointestinal Side Effects**

Although immunotherapy is not nearly as emetogenic as chemotherapy, some patients still may experience nausea and vomiting of unclear etiology. The use of antiemetics such as a serotonin receptor antagonist may help with the nausea.

Although the use of narcotics more commonly induces constipation, diarrhea may also be seen during immunotherapy. Although constipation may be related to dinutuximab and/or cytokines, the possibility of infection should be considered. It is our practice that if a patient is symptomatic, stool cultures are sent. Despite careful infection-control procedures and judicious use of antibiotics, up to 20% of our patients have experienced *Clostridium difficile* infection at some point during immunotherapy. As a result, we have a high index of suspicion in patients who develop diarrhea during or after an admission for dinutuximab therapy.

## USE OF ISOTRETINOIN

A 14-day course of isotretinoin is included in each cycle of dinutuximab therapy. Careful administration of this medication is important, as it affects absorption. The administration of isotretinoin to the younger patient population in particular can lead to challenges. The medication is only commercially available in capsules. Our child life staff has been instrumental in teaching younger patients how to swallow capsules. If pill swallowing still remains a barrier, we have worked with our pharmacy staff to develop ways to effectively administer the medication. Although swallowing pills remains the best option, many patients have been able to chew the capsules. As a last resort, our nurse practitioner staff has been able to work with families to puncture and squeeze the contents into high-fat foods such as peanut butter or ice cream immediately before administration. We strongly discourage administering the medication through enteral tubes, because there is concern about physical medication adherence to the tube.

Isotretinoin is associated with its own side effects. Two of the most common side effects seen in our experience are elevated liver function tests and skin toxicity. Prior to each course of isotretinoin, liver function tests, including triglycerides, should be checked. Patients often develop very dry skin and cheilitis. These side effects are often managed well with moisturizers such as Aquaphor and topical vitamin E and typically improve quickly after the course of isotretinoin is complete. Less frequently, some of our patients have developed photosensitivity and difficulty seeing in the dark. These side effects usually resolve after the medication is completed.

## ROLE OF THE ADVANCED PRACTITIONER

As one can see, the benefits of immunotherapy do not come without risks. Having front-line providers who are familiar with immunotherapy and its associated toxicities is critical to the safe and consistent administration of this complex therapy. These front-line providers can include physicians, nurse practitioners, physician assistants, and bedside nurses. The Children’s Hospital of Philadelphia utilizes mainly nurse practitioners as the front-line ordering clinicians caring for this patient population. This approach permits consistency of care, as attending physicians, fellows, and residents often rotate.

Furthermore, nurse practitioners in our institution have developed easy-to-follow algorithms for managing common side effects. The algorithms clearly delineate a standard approach to common and potentially serious side effects seen in patients with high-risk neuroblastoma receiving dinutuximab-based immunotherapy. With these algorithms in place, practices are consistent among front-line providers, and families are given a consistent plan for toxicity management. Appendices A to E provide examples of the algorithms that front-line clinicians use in practice at the Children’s Hospital of Philadelphia.

**Appendix A.**
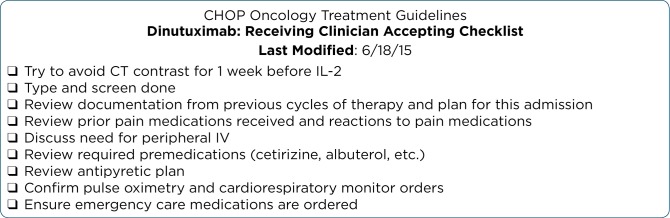
Appendix A. Dinutuximab Algorithm: Receiving Clinician Accepting Checklist

**Appendix B.**
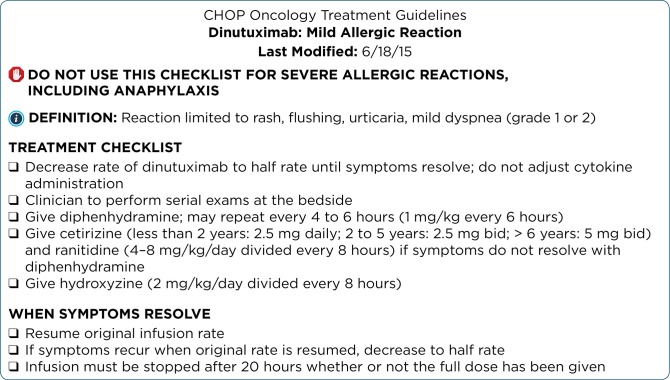
Appendix B. Dinutuximab Algorithm: Mild Allergic Reaction

**Appendix C.**
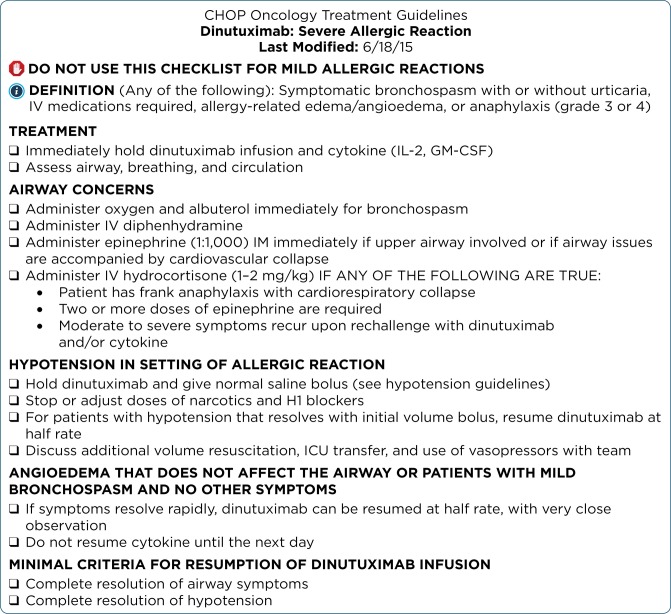
Appendix C. Dinutuximab Algorithm: Severe Allergic Reaction

**Appendix D.**
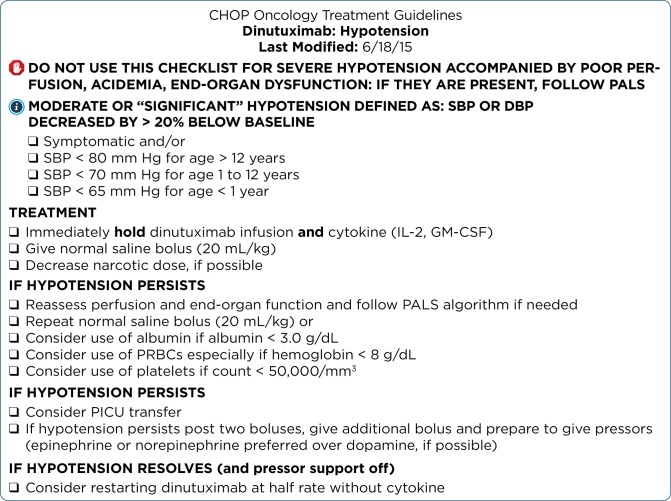
Appendix D. Dinutuximab Algorithm: Hypotension

**Appendix E:**
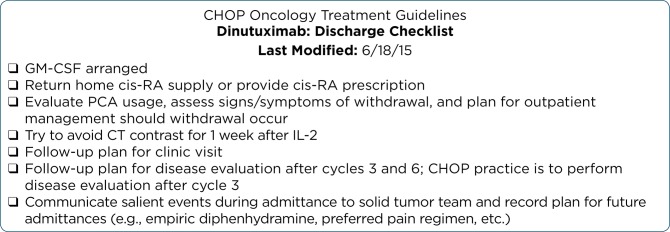
Appendix E. Dinutuximab Algorithm: Discharge Checklist

Nurse practitioners have also been integral in the education of bedside nurses who care for children receiving immunotherapy. As a result of educational interventions, nurses are fully aware of the expected side effects of therapy and are confident in their ability to recognize toxicities before they become severe. Bedside nurses can follow the algorithms and contact front-line providers when appropriate. Consistency and reinforcement of standard practices have decreased staff anxiety over delivery of care, which in turn has decreased patient and parental anxiety as well.

It is essential to have experienced front-line providers in the outpatient as well as inpatient setting. The outpatient provider must educate families regarding the medications that are included in the immunotherapy cycles and must explain the expected side effects. Managing the expectations of families is critically important, and the more knowledgeable parents/guardians are, the smoother the transition to immunotherapy will be.

For example, for a child about to begin course 1 (dinutuximab and GM-CSF), parents should expect that day 1 is going to be very difficult. Parents should be told that their child will experience fevers, will look puffy, and will gain weight. On day 1, the child will likely be in pain until the PCA can be titrated to effect. Informed parents can then decide how best to handle the time during which their child will experience pain. The outpatient provider can also coach parents to be the eyes and ears of the staff. Our experience is that when parents are optimally educated, they are generally the first to detect hives or rashes, and their help in identifying treatable allergic reactions early leads to improved care.

Although the same patient may experience different toxicities during each immunotherapy cycle, the outpatient provider can speak to the family about what toxicity management strategies worked best for specific toxicities. This information can be used to adjust the plan of care for future cycles, with the goal to improve symptom management and safely deliver immunotherapy.

The safety and quality of care during immunotherapy are also impacted by the level of communication between inpatient and outpatient providers. Feedback from inpatient providers regarding the effects experienced and required adjustments in medications is critical to preparation for the next admission. The outpatient provider can then prepare accordingly and ensure that the orders for subsequent cycles are appropriate, so side effects can be minimized. If a patient developed hives on day 1 of a dinutuximab infusion and additional antihistamines were effective, additional antihistamines should be incorporated into orders for future admissions. Consistency of care and ongoing communication are essential.

## CONCLUSION

The addition of dinutuximab, cytokines, and isotretinoin to multimodality therapy for children with high-risk neuroblastoma has improved event-free survival rates (Yu et al., 2010). However, toxicities associated with this therapy are significant. The leadership of front-line providers in taking a systematic approach to managing the most common side effects and potentially severe toxicities is important. Advanced practitioners can develop tremendous expertise in caring for these patients. Care for children receiving immunotherapy is improved when front-line providers are consistent in their management of side effects and in their ongoing education of bedside staff and families.

**Acknowledgments**

We thank Ro Bagatell, MD, for her support, encouragement, and assistance in the care of these patients and with the development of this article. We also thank Naomi Balamuth, MD, for her help in the development of our treatment guidelines and flow diagrams; and Carol Tustin, our data manager, for her time and assistance in collecting and providing requested data on our patients. We are grateful to the oncology nurses who provide such wonderful expert care to this population of patients. We also acknowledge and thank the ICU staff for the successful collaborative care of those patients requiring ICU transfer.
